# ProteoMapper: Alignment-aware identification and quantitative analysis of contextual motif–domain patterns in protein families

**DOI:** 10.1371/journal.pone.0348861

**Published:** 2026-08-10

**Authors:** Sifullah Mahmud Sefa, Joyeeta Sarkar, Arif Hasan Khan Robin, Machbah Uddin

**Affiliations:** 1 Department of Computer Science and Mathematics, Bioinformatics Engineering, Bangladesh Agricultural University, Mymensingh, Bangladesh; 2 Department of Genetics and Plant Breeding, Bangladesh Agricultural University, Mymensingh, Bangladesh; Irkutsk State University: Irkutskij gosudarstvennyj universitet, RUSSIAN FEDERATION

## Abstract

Understanding how sequence patterns relate to conserved protein domains is fundamental for interpreting protein function and organization. Although numerous tools support motif detection and domain annotation, assessing their positional relationships across multiple sequence alignments typically requires combining outputs from separate analyses. Here, we present *ProteoMapper*, a Python-based toolkit for alignment-aware annotation and visualization of protein sequences. The framework integrates user-defined regular expression–based motif detection with HMMER-based domain annotation using Pfam profiles, and maps both features directly onto aligned sequences within a unified system. Results are exported as a multi-sheet spreadsheet with standardized visual annotations, enabling simultaneous inspection of motif occurrences, domain regions, and positional patterns across homologous proteins. ProteoMapper additionally computes a motif–domain coverage score (MDCS), defined as the fraction of motif residues overlapping annotated domains. This metric provides a descriptive measure of motif localization relative to domain regions and supports differentiation between domain-associated sequence signatures and motifs occurring outside annotated domains. To complement this, the tool reports positional conservation of motifs across alignments, facilitating identification of positionally constrained sequence patterns. The utility of ProteoMapper is demonstrated through case studies including validation of domain detection against published datasets, analysis of conserved sequence signatures in ERD6-like sugar transporter proteins, and evaluation of regulatory motif localization in HIF-1α sequences. ProteoMapper provides an integrated and accessible framework for visualization and basic quantification of motif and domain annotations within an alignment context, supporting exploratory analysis of protein sequence organization. Source code, documentation, and datasets are available at https://github.com/sifullah0/ProteoMapper.

## Introduction

The functional characterization of proteins relies on understanding how conserved sequence features relate to structural domains and regulatory elements. Protein domains represent evolutionary conserved units associated with specific biochemical functions, and are commonly identified using profile hidden Markov models (HMMs) implemented in tools such as HMMER [1] and databases such as Pfam [2]. Although, predicted domain regions may include partially disordered segments and do not always correspond to fully structured units. In parallel, sequence motifs are defined broadly as recurring amino acid patterns and widely used to annotate functional sites, including enzyme signatures, binding regions, and post-translational modification sites [3].

It is important to distinguish between different classes of motifs. Some motifs correspond to conserved sequence signatures embedded within structured domains, such as PROSITE patterns, and often reflect domain-specific sequence constraints. In contrast, short linear motifs (SLiMs) represent a distinct class of functional elements that are typically short, degenerate, and located in intrinsically disordered regions, where they mediate transient interactions or regulatory modifications [4]. These different motif types exhibit fundamentally different positional and structural properties, and their interpretation requires careful contextualization. It is important to note that computational identification of SLiMs requires convergent evidence from structural context (location in accessible regions), functional characterization (demonstration of regulatory activity), and evolutionary analysis (conservation across orthologs).

Motif searching and pattern detection can be performed using tools such as MEME Suite [5] and its associated components (e.g., FIMO, STREME [6,7]), while integrated annotation platforms such as InterProScan [8] combine multiple domain and motif resources. Additionally, resources such as the ELM database provide curated SLiM annotations alongside domain information [9]. These tools offer sophisticated capabilities for identifying sequence features, but often present motif and domain annotations separately or require multiple steps to relate them within a common alignment context.

Regular expression–based pattern matching remains a widely used approach for detecting predefined sequence motifs, particularly short linear motifs (SLiMs) and domain-associated sequence signatures. Resources such as the ELM database [9–11] and ScanProsite [12] rely on regular expression–based definitions for motif annotation. In this context, the use of regular expressions in ProteoMapper is not intended for de novo motif discovery, but to enable flexible, user-defined detection of known or hypothesis-driven sequence patterns within an alignment-aware framework.

Despite the availability of these resources, examining the positional relationships between sequence patterns and domain annotations across a multiple sequence alignment remains a largely manual process. In particular, assessing whether a motif consistently occurs within, outside, or at the boundaries of annotated domains across homologous sequences can require integrating outputs from multiple tools and formats. This can be especially relevant when comparing domain-associated sequence signatures with regulatory motifs that are expected to occur in distinct structural contexts.

Here, we present *ProteoMapper*, a Python-based tool designed for alignment-aware annotation and visualization of protein sequences. ProteoMapper integrates four core components: (i) regular expression-based detection of user-defined sequence patterns, (ii) domain annotation using HMMER against Pfam profiles, (iii) post-detection analyses including positional conservation and motif-domain context assessment, and (iv) generation of an alignment-aware spreadsheet output that simultaneously maps motif matches and domain regions onto a unified coordinate system. This integrated representation allows motif occurrences, domain annotations, and conservation patterns to be examined within the same alignment context.

An additional feature of ProteoMapper is the computation of a simple motif-domain coverage score (MDCS), defined as the fraction of motif residues overlapping annotated domain regions. This metric provides a descriptive measure of whether a given sequence pattern tends to occur within annotated domains or outside them across a dataset. While this score does not imply functional interaction or structural coupling, it can aid in distinguishing domain-associated sequence signatures from motifs that are more likely to occur in non-domain regions. In this context, MDCS may help highlight candidate motifs for further investigation, including those consistent with short linear motifs (SLiMs) or other regulatory sequence features, when interpreted alongside existing biological knowledge.

The utility of ProteoMapper is demonstrated through case studies involving (i) validation of domain detection against published datasets, (ii) analysis of conserved sequence signatures within transporter domains, and (iii) evaluation of regulatory motif localization in HIF-1α, where known short linear motifs are predominantly located outside structured domains. Together, these examples illustrate how alignment-aware integration of sequence features can support exploratory analysis of protein sequence organization.

ProteoMapper is not intended to replace established tools for motif discovery or domain prediction. Instead, its primary contribution lies in facilitating the combined visualization and basic quantification of sequence features within an alignment context. By providing an integrated representation of motif occurrences, domain annotations, and positional conservation, the tool enables users to examine positional relationships between sequence features in an interpretable biological context.

It should also be noted that domain boundaries reported by ProteoMapper are derived from sequence-based HMM matches and therefore represent approximate domain extents. These boundaries do not necessarily correspond to precise structural domain definitions, which are more accurately determined using three-dimensional structural information or structure-based domain parsing methods [13,14].

## Materials and methods

**Fig 1:**
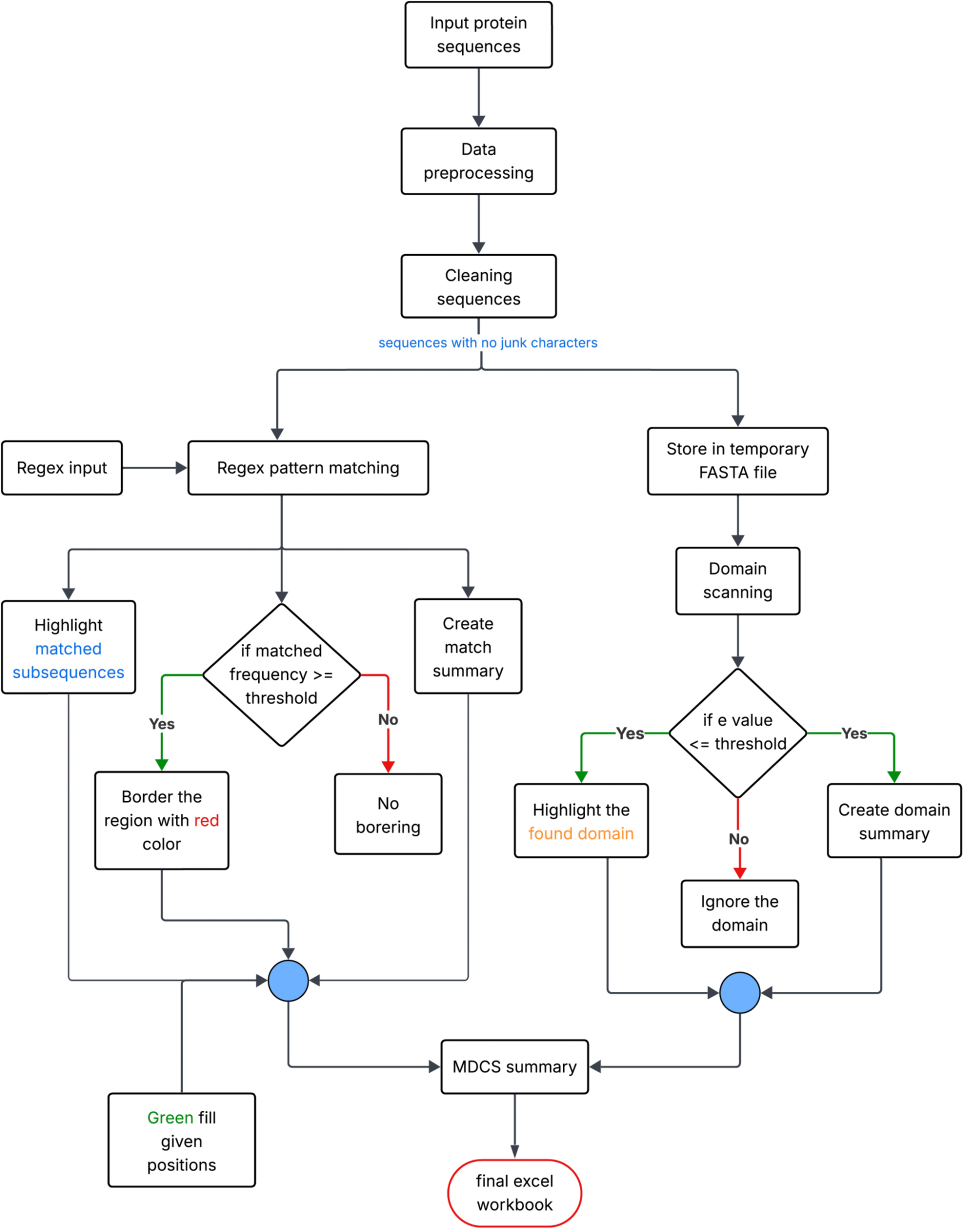
Workflow of the ProteoMapper sequence-analysis pipeline. Input preprocessing branches into motif matching and HMMER/Pfam domain scanning before results are integrated into the final Excel workbook.

In this section, various prerequisites are discussed, including dataset collection and preparation, the main algorithms, and the necessary tools for software development. The general workflow of the toolkit is presented in Fig 1.

### Data collection and preprocessing

ProteoMapper accepts aligned protein sequences in either Excel format (.xlsx, .xls) or FASTA format (.fasta, .fa, .faa). FASTA input provides compatibility with standard sequence-analysis workflows, while Excel input is retained as an accessibility option for users who prefer to inspect sequence identifiers, metadata, and aligned residues in a single tabular file.

For Excel input, the file must contain a protein sequence column and at least one supported identifier column, such as Gene Name, Gene ID, Protein Name, or Protein ID. To support user-generated spreadsheets with variable layouts, ProteoMapper detects the header row dynamically by scanning for these required column names. FASTA records are parsed into the same internal tabular structure used for Excel input, allowing both input formats to follow a common downstream workflow.

A key preprocessing step in ProteoMapper is gap-aware normalization. For each sequence, two synchronized representations are generated. The first is a display view, which preserves alignment gaps (-) for visualization and inspection of multiple sequence alignments. The second is a matching view, in which gaps and non-amino-acid characters are removed to produce a contiguous sequence for regex-based motif detection and HMMER/Pfam domain annotation. Motif and domain coordinates detected in the gapless matching view are then mapped back to the gapped display view, allowing annotations to be visualized in the original alignment context. This approach avoids motif-search artifacts caused by alignment gaps while preserving the biological interpretability of alignment-based positional relationships.

Input cleaning also includes basic handling of malformed or incomplete sequence entries. Whitespace, line breaks, unsupported characters, and formatting artifacts are removed before analysis, and missing sequence values are handled as empty strings to avoid downstream runtime errors. These steps ensure that motif detection and domain scanning are performed on standardized sequence representations while keeping the original aligned layout available for output visualization.

Because spreadsheet software can automatically alter some gene names or identifiers, ProteoMapper includes safeguards to reduce Excel-related data corruption during import. Excel fields are read as text, and the software does not intentionally perform automatic date, numeric, or formula-based conversion of identifier columns. Identifier columns are preserved during preprocessing and are not used for coordinate calculations. However, if a gene name or identifier has already been converted by spreadsheet software before the file is imported into ProteoMapper, the original value may not be recoverable automatically. Therefore, users are advised to format identifier columns as text before entering data, verify identifiers before analysis, or use FASTA input when identifier integrity is critical.

### Pattern matching and highlighting

This toolkit supports flexible detection of protein motifs using user-defined regular expressions. Unlike fixed pattern-matching methods, the regex engine allows user-defined specification of motif patterns. Users may define specific or degenerate motifs, such as phosphorylation sites (e.g., [ST]..[DE]) or family-specific signatures that are not present in standard databases. Modification sites such as N-glycosylation ((N[Pˆ][ST][Pˆ]) can be identified as well. Patterns are entered directly through the graphical interface (GUI); the current release does not provide a command-line option for specifying motif patterns.

The motif-matching engine operates in two modes. For datasets with fewer than 100 sequences, the system uses serial execution. For larger datasets, computations are distributed across multiple worker processes. Users can set the number of processes directly through the interface. Complete algorithmic details are provided in Algorithm 1.

#### Conservation threshold and positional constraint.

Users can specify the minimum percentage of sequences that must contain a given motif at identical alignment coordinates for that motif to be classified as positionally conserved. The default threshold is 60%, though this value is fully user-adjustable through the interface. As with other conventional thresholds used in sequence analysis, such as E-value cutoffs in profile-HMM searches or bootstrap support values in phylogenetic inference, the appropriate stringency depends on the size and evolutionary divergence of the dataset and is therefore left to the user’s discretion. We illustrate this flexibility in practice: whereas the default 60% is used in the main case studies, Case Study-2 applies a 55% threshold reflecting its smaller dataset (10 of 17 sequences required for conservation). Motifs that meet or exceed this threshold are highlighted in the output with a red border, indicating evolutionary constraint on motif placement. This threshold-based classification distinguishes motifs that are positionally fixed from those that are positionally variable, and should be treated as a configurable screening criterion rather than a claim of biological significance.

To quantify the degree of positional variability, ProteoMapper calculates the positional dispersion (σ) for each detected motif pattern. Positional dispersion is calculated as the standard deviation of starting positions of each motif. A value of σ = 0 indicates that the motif appears at a single, invariant position in all sequences containing it, reflecting strict positional conservation. Higher σ values indicate greater positional variability, suggesting that the motif is tolerated at multiple locations. This facilitates identification of recurrent or conserved motif regions and quantifies the degree of positional constraint for each pattern.

**Algorithm 1: Motif matching and conservation highlighting**



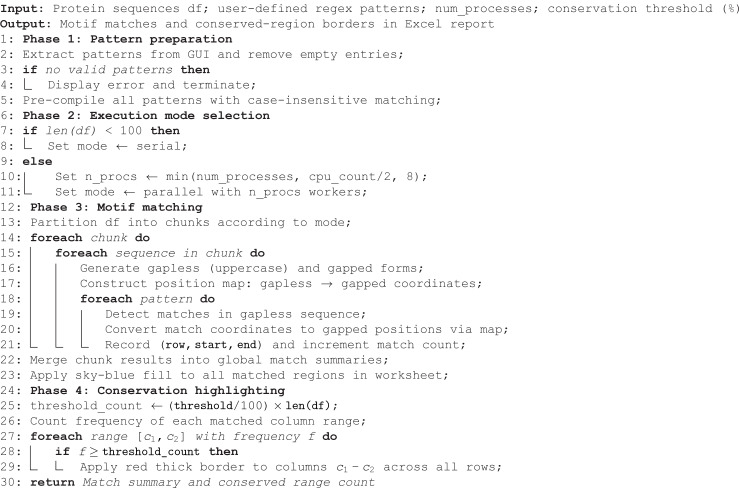



#### Excel visualization and error handling.

Results are presented in a multi-sheet Excel workbook with standardized visual annotations to facilitate interpretation. Detected motif occurrences are highlighted using a **sky blue fill**, while motifs meeting the user-defined conservation threshold are marked with a thick **red border** across aligned positions. User-specified alignment columns can be optionally highlighted with a **green fill** to enable targeted inspection of known functional or variant sites. The workflow incorporates basic validation and error-handling procedures to ensure robust execution. Invalid regular expression patterns are detected and reported prior to analysis. Alignment gaps (“-”) are ignored during motif matching to prevent positional artifacts, while coordinate mapping ensures accurate projection of matches onto the gapped alignment. In addition, edge cases such as empty sequences or malformed inputs are handled gracefully to avoid runtime errors.

### Conserved domain prediction using HMMER

The pipeline can process large sequence sets more efficiently through parallel execution. In this mode, multiple hmmscan processes run simultaneously. Each process scans a different subset of the input sequences. The results from all processes are then combined into a single output file. First, input sequences are cleaned (gaps removed) and saved to a temporary FASTA file. Each sequence is assigned a numeric ID that maps to its original alignment position for full traceability. Then, hmmscan is executed with the domtblout output format to perform sequence–profile alignments against Pfam-A. The domain boundaries reported here are sequence-based approximations derived from HMMER profile matches and do not necessarily correspond to precise structural domain boundaries defined by three-dimensional protein structure. The pipeline performs error checks to detect missing executables or execution failures. Then, hits are filtered to retain only high-confidence matches (independent E-value ≤0.001). Parsed fields include query ID, Pfam accession (e.g., PF00071), domain start/end positions, and the bit score. Finally, structured results are written to hitdata.txt with consistent delimiters. The pseudocode for all of these processes is presented in Algorithm 2. Full domain descriptions are preserved, and E-values are presented in scientific notation for accuracy. The output format is designed to support reproducibility and traceability to the original alignment positions.

**Algorithm 2: Parallel domain detection using multiple hmmscan instances**



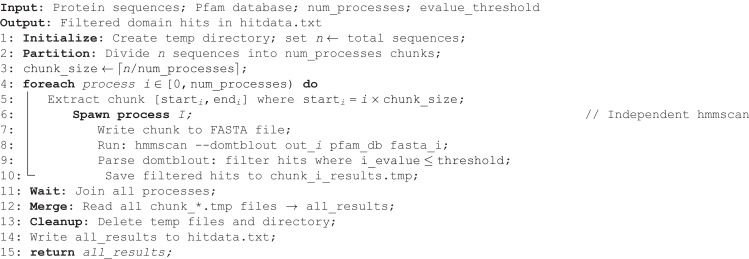



#### Domain highlighting in Excel.

The pipeline generates annotated domain visualisations in Excel through automated formatting and metadata embedding. Detected domain boundaries are marked using an orange cell fill applied to all residue positions within the predicted domain span which is presented in Algorithm 3. In case of embedded metadata, dynamic cell comments are generated for each domain’s start position, containing:

- Domain name: *description*
- Accession: *accession*
- Coords: *start-end*
- Uses openpyxl’s PatternFill with exact RGB color matching
- Implemented coordinates(start-end positions) in the comment are not according to HMMER’s gap-free sequence numbering. They are calculated based on aligned protein sequences(contains ’-’ in sequences)
- Skips invalid positions (start > end or negative values)

**Algorithm 3 Domain highlighting in an Excel worksheet**



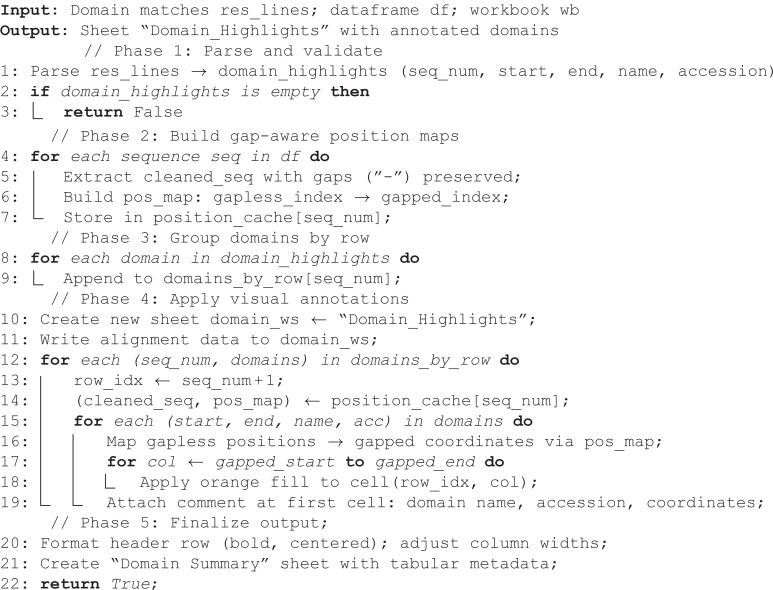



### Motif–domain coverage score (MDCS)

Motif detection and domain annotation are commonly performed as independent analyses. Yet the positional relationship between short linear motifs and conserved domains in protein sequences can provide important biological insights. To support this interpretation, ProteoMapper computes the Motif–Domain Coverage Score (MDCS), a quantitative measure describing the spatial relationship between motifs and predicted protein domains.

For each motif occurrence and each overlapping Pfam domain within the same sequence, MDCS is defined as the proportion of the motif length that overlaps the domain:


$$\begin{array}{c} \text{MDCS}=\frac{\text{length of motif–domain overlap}}{\text{motif length}} \end{array}$$

MDCS values range from 0 to 1. A value of 1 indicates that the motif is fully contained within a domain, values between 0 and 1 indicate partial overlap with domain boundaries, and a value of 0 indicates that the motif lies outside annotated domains. When a motif overlaps a domain multiple times within a sequence, ProteoMapper retains the highest MDCS value observed for the domain accession to summarize the strongest motif–domain association. MDCS calculation is performed using gapless sequence coordinates derived from domain predictions to ensure consistency with Pfam annotations.

The results are reported in a dedicated MDCS Summary worksheet containing three complementary tables. The first table lists per-sequence details including sequence index, motif pattern, associated domain accession(s), MDCS values, and a qualitative interpretation (fully embedded, partial overlap, or outside domains). The second table summarizes domain-specific motif embedding patterns. It reports the percentage of occurrences where each motif is fully or partially embedded within each domain, along with the total count of motif–domain overlaps. This identifies which domains are frequently associated with specific motif patterns. Domains showing high motif overlap counts may highlight regions of potential interest for further investigation. The third table provides motif-level statistics, including mean and median MDCS values of each motif calculated from the highest MDCS per domain per sequence, total occurrence counts, occurrences touching domains versus those completely outside all domains. Together, these statistics enable users to distinguish motifs that are consistently embedded within structured domains from those occurring primarily in disordered regions or at domain boundaries, complementing motif conservation and frequency analyses without adding substantial computational overhead.

### Software and tools used

ProteoMapper integrates Python 3.10 with specialized libraries for protein analysis. Python packages such as, Pandas was used for data handling, NumPy was used for numerical operations, and openpyxl was used for Excel formatting. Domain detection employs HMMER v3.4 (hmmscan) with the Pfam-A.hmm v37.0 database, providing comprehensive domain annotation capabilities. Pfam-A.hmm v37.0 was the most recent release available at the time of analysis. A subsequent release (v38.2) has since become available; as this update does not represent a major revision of the well-characterized domain families examined in this study (e.g., PLATZ, ADF-H, and MFS transporter domains), the reported domain annotations were not regenerated with the newer release.

### Use of AI tools in manuscript preparation

During preparation of this manuscript, the authors used ChatGPT and Claude for language refinement, grammar correction, and improvement of clarity in the manuscript text. These tools were not used for study design, data generation, or the production of any reported results, figures, or conclusions. All AI-assisted text was reviewed properly to verify its accuracy.

## Results

This section presents the ProteoMapper graphical interface, the structure of its multi-sheet output, three case studies assessing domain-detection accuracy and motif–domain relationships, and a benchmark of computational performance.

### User interface

**Fig 2:**
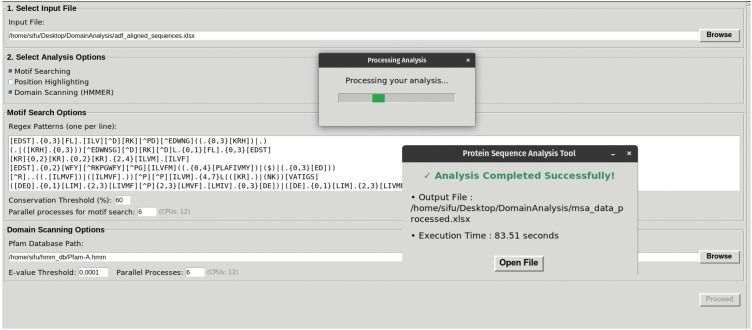
ProteoMapper graphical user interface. The interface shows input selection, motif-search options, domain-scanning settings, processing status, and completion output.

After running the project code, the interface shown in Fig 2 is displayed, providing an intuitive platform for users to input protein sequence alignments, configure parameters (e.g., regex patterns, conservation thresholds, and HMMER options), and visualize results. The interface allows real-time preview of motif matches, domain annotations, and user-defined highlights.

### Dataset and toolkit output structure

**Fig 3:**
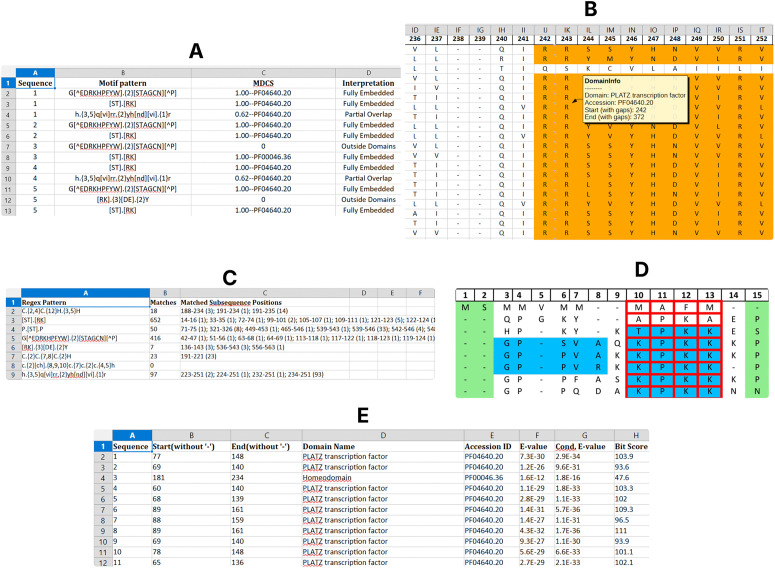
Representative ProteoMapper output sheets. (A) MDCS_Summary illustrating motif–domain embedding relationships; (B) Domain_Highlights with orange Pfam domains and embedded metadata; (C) Match_Summary; (D) First_Sheet with sky-blue motif matches and red borders on conserved columns; (E) Domain_Summary containing HMMER statistics.

ProteoMapper was evaluated using validated biological datasets: (i) PLATZ transcription factor sequences from multiple plant species [15,16], (ii) Actin-depolymerizing factor (ADF/cofilin) proteins [17,18], (iii) ERD6-like (EDL) sugar transporter proteins from *Arabidopsis thaliana* [19,20], and (iv) HIF-1α proteins from vertebrate species for regulatory motif analysis. Each dataset was provided as a gapped multiple-sequence alignment. ProteoMapper accepts alignments in both Excel format and FASTA format. After processing, ProteoMapper produces a single multi-sheet Excel workbook that consolidates all analyses (Fig. 3). The structure of the output is described below.

- **First_Sheet:** Contains the complete alignment matrix. Regex-based motif matches are shown in **sky-blue**. Columns where a motif appears in ≥60% (threshold in this test) of sequences (user-adjustable) receive a **thick red border**, highlighting highly conserved motif positions without manual inspection (Fig 3D).
- **User-defined position highlighting:** Any alignment columns specified by the user (space-separated numbers) are filled bright green across all rows, enabling rapid visual mapping of known functional or variant sites (Fig 3D).
- **Match Summary:** An automatically generated table listing each user-defined regex pattern, its total match count, positional spans detected in the alignment, and column-wise conservation statistics. Positional dispersion (σ) is also reported for each motif, quantifying the degree of positional variability across sequences (Fig 3C).
- **Domain_Highlights:** A duplicate of the alignment with Pfam domains obtained from parallel hmmscan scans (E-value ≤0.001 for this test). Detected domains are displayed in **orange**. Each domain block includes an embedded Excel comment containing the domain name, accession ID, bit score, E-value, and conditional E-value, providing complete HMMER metadata directly within the sheet (Fig 3B).
- **Domain Summary:** A tabular summary of all detected domains, including E-values, conditional E-values, and bit scores. E-values represent the expected number of false positives, with lower values indicating higher confidence, while bit scores provide a log-scaled measure of match quality (Fig 3E).
- **MDCS Summary:** A three-table summary quantifying motif–domain relationships. The first table reports per-sequence MDCS values for each motif occurrence, listing the highest MDCS value observed for each overlapping Pfam domain (identified by accession ID) together with a qualitative interpretation (fully embedded, partial overlap, or outside domains). The second table summarizes domain-specific motif embedding patterns, reporting the percentage of occurrences where each motif is fully or partially embedded within each domain, along with total overlap counts. The third table provides motif-level statistics, including mean and median MDCS values, total occurrence counts, and separate tallies of occurrences overlapping domains versus those completely outside all domains (Fig 3A).
- **hitdata.txt:** A plain-text file containing all raw hmmscan hits, supporting downstream filtering, custom parsing, or archival of domain prediction results with complete metadata.

This unified Excel-based structure enables immediate comparison of motif occurrences, positional conservation, user-specified functional sites, conserved domain boundaries, and motif–domain embedding relationships. Processed output files for all datasets are available at https://github.com/sifullah0/ProteoMapper/tree/main/sample_datasets%26outputs.

### Case study 1: Validation of domain detection on *BrPLATZ* and *SlADF* proteins

The accuracy of ProteoMapper’s domain detection was assessed by comparison with two previously published datasets for which domain annotations were independently established.

The 24 PLATZ transcription factor sequences from *Brassica rapa* (BrPLATZ1–BrPLATZ24) reported by Azim et al. [16], originally annotated using the SMART database [21], were used as a reference dataset. Application of hmmscan against Pfam-A (E-value ≤0.0001) reproduced 22 of 23 reported PLATZ domain (PF04640) spans exactly, presented in Table 1. BrPLATZ17 returned a truncated match (74–98 vs. reported 74–127), likely reflecting partial domain recovery or reduced sequence similarity near the C-terminal region. No PLATZ domain was detected in BrPLATZ3 in either the original study or the present analysis. The homeobox domain (PF00046) reported in BrPLATZ3 was recovered at residues 181–234, closely matching the published range of 180–235. Bit scores for PLATZ domain hits ranged from 28.3 to 111.0, with all E-values $\leq 7.3\times 10^{-6}$, confirming the statistical significance of detections. SMART reported B-box zinc finger domains (PF00643) in 15 of 24 proteins, which were not detected under the applied E-value threshold. This discrepancy reflects differences between SMART and Pfam hidden Markov models for the same domain family [21]. It is consistent with published PLATZ studies that do not treat the B-box as a primary functional region [22].

**Table 1:** Comparison of PLATZ and homeobox domain ranges. Domain ranges detected by SMART [16] and ProteoMapper using Pfam/HMMER.

Protein	PLATZ (Paper)	PLATZ (Tool)	Homeobox (Paper)	Homeobox (Tool)	Match Type
BrPLATZ1	77–148	77–148			Exact
BrPLATZ2	69–140	69–140			Exact
BrPLATZ3			180–235	181–234	Partial
BrPLATZ4	60–140	60–140			Exact
BrPLATZ5	68–139	68–139			Exact
BrPLATZ6	89–161	89–161			Exact
BrPLATZ7	88–159	88–159			Exact
BrPLATZ8	89–161	89–161			Exact
BrPLATZ9	69–140	69–140			Exact
BrPLATZ10	78–148	78–148			Exact
BrPLATZ11	65–136	65–136			Exact
BrPLATZ12	68–139	68–139			Exact
BrPLATZ13	90–164	90–164			Exact
BrPLATZ14	89–161	89–161			Exact
BrPLATZ15	84–158	84–158			Exact
BrPLATZ16	90–164	90–164			Exact
BrPLATZ17	74–127	74–98			Partial
BrPLATZ18	133–204	133–204			Exact
BrPLATZ19	88–160	88–160			Exact
BrPLATZ20	69–140	69–140			Exact
BrPLATZ21	60–141	60–141			Exact
BrPLATZ22	67–138	67–138			Exact
BrPLATZ23	66–137	66–137			Exact
BrPLATZ24	73–143	73–143			Exact

The 11 actin-depolymerizing factor (ADF) sequences from *Solanum lycopersicum* characterised by Khatun et al. [18], in which the ADF-H domain (PF00241) was confirmed via SMART and InterPro, were used as a second reference dataset. The ADF-H domain was detected in all 11 proteins with high confidence, shown in Table 2.

**Table 2:** Comparison of ADF-H domain boundaries in tomato SlADF proteins.

Protein	Published span	ProteoMapper span	IoU
SlADF1	12–137	7–132	0.93
SlADF2	16–143	12–142	0.94
SlADF3	12–137	8–135	0.94
SlADF4	12–139	7–137	0.93
SlADF5	12–137	7–136	0.96
SlADF6	12–137	7–135	0.94
SlADF7	12–139	7–138	0.96
SlADF8	18–145	14–145	0.96
SlADF9	18–145	13–143	0.93
SlADF10	12–139	7–138	0.96
SlADF11	12–139	7–138	0.96

Boundary agreement was quantified using Intersection-over-Union (IoU):


$$\begin{array}{c} \text{IoU}=\frac{\min({\text{end}}_{\text{tool}},{\text{end}}_{\text{paper}})-\max({\text{start}}_{\text{tool}},{\text{start}}_{\text{paper}})+1}{\max({\text{end}}_{\text{tool}},{\text{end}}_{\text{paper}})-\min({\text{start}}_{\text{tool}},{\text{start}}_{\text{paper}})+1} \end{array}$$

Mean IoU was 0.94 (range 0.93–0.96), indicating greater than 90% positional overlap in all cases. Start positions were, on average, 4.8 residues earlier than those in the published annotation, a systematic offset consistent with previously reported differences between Pfam HMMs and SMART.

Across both datasets, domain detection by ProteoMapper showed close agreement with independently established annotations, with complete recovery of expected domains in both cases. Minor boundary differences are likely attributable to known methodological distinctions between Pfam and SMART model curation. These results indicate that the domain annotation component of ProteoMapper produces reliable output and the detected domain coordinates are suitable for subsequent motif–domain spatial analyses.

### Case study 2: Conservation and domain-context analysis of sugar transporter signatures in ERD6-like proteins

ERD6 (Early Responsive to Dehydration 6) was originally identified in *Arabidopsis thaliana* as a stress-induced gene encoding a putative sugar transporter [19]. Subsequent phylogenetic analyses have shown that ERD6-like (EDL) proteins form a conserved subfamily of sugar transporters in land plants, exhibiting lineage-specific sequence variation [20]. These proteins are annotated as members of the Major Facilitator Superfamily (MFS), and PROSITE patterns PS00216 and PS00217 have historically been used as sequence signatures associated with this transporter class [3].

Seventeen EDL proteins from *A. thaliana* (UniProt entries) were aligned and analysed using ProteoMapper. PROSITE-derived regular expressions corresponding to PS00216 and PS00217 were used as search patterns. Domain annotation was performed using HMMER against the Pfam-A database (E-value ≤0.0001), and positional conservation was assessed using a threshold of 55%, requiring a pattern to occur at the same aligned position in at least 10 of 17 sequences.

HMMER detected the Sugar (and other) transporter domain (PF00083.31) in all sequences (E-values ranging from 10^−53^ to 10^−101^), confirming family membership. The broader MFS domain (PF07690.22) was also detected in all sequences as a secondary annotation. Both domains span the majority of each protein (approximately residues 27–487), consistent with the compact transmembrane architecture characteristic of MFS transporters [23].

PS00217 was detected in 10 of 17 sequences (58.8%), exclusively at a single aligned region (positions 171–196; positional dispersion σ=0.0). This pattern met the positional conservation threshold and was classified as positionally conserved. All instances were fully embedded within both PF00083.31 and PF07690.22 domains (MDCS = 1.0).

PS00216 was detected in 13 sequences, distributed across two distinct alignment regions, at positions 129–146 (6 sequences, 35.3%) and positions 363–381 (7 sequences, 41.2%). Neither region independently satisfied the positional conservation threshold, and the pattern was therefore not classified as positionally conserved. All detected instances were fully embedded within annotated domains (MDCS = 1.0).

These results highlight distinct positional behaviours of two established MFS-associated sequence patterns within the same protein family. Both PS00216 and PS00217 are fully contained within transmembrane domains, consistent with domain-associated sequence features rather than short linear motifs typically found in disordered regions [12]. The strict positional conservation of PS00217 suggests strong evolutionary constraint, potentially reflecting structural or functional importance within the transporter fold. In contrast, the positional variability of PS00216 across two non-overlapping regions may reflect sequence divergence within the EDL family, consistent with previously reported lineage-specific variation [20].

Overall, this analysis illustrates how the integration of positional conservation and domain-context scoring can distinguish conserved, positionally constrained patterns from more variable sequence features within protein domains.

### Case study 3: Domain-context analysis of HIF-1α regulatory motifs

HIF-1α is the oxygen-sensitive subunit of the HIF-1 transcription factor and a central regulator of the cellular hypoxic response. Its regulation is mediated through multiple post-translational modifications and protein–protein interactions that occur predominantly within intrinsically disordered regions (IDRs), including the oxygen-dependent degradation domain (ODD) and transactivation domains (TADs) [24,25]. Such regions are well known to host short linear motifs (SLiMs), which function as modular interaction and modification sites in flexible sequence contexts [4].

To evaluate whether motif–domain overlap reflects this established biology, nine motif patterns derived from ELM resource [9,26] were analysed across a multiple sequence alignment of 37 vertebrate HIF-1α orthologues. Six of these motif classes correspond to experimentally studied regulatory mechanisms reported in the literature, including phosphorylation, SUMOylation, and protein–protein interaction sites [27–32]. These motif classes are treated as consensus representations of experimentally characterized functional regions rather than exact residue-level annotations.

Motif instances were mapped onto Pfam domain annotations obtained using HMMER (E-value $\leq 1.0\times 10^{-3}$), and the motif–domain context score (MDCS) was computed as the fraction of motif residues overlapping annotated domains.

MDCS is a continuous measure of spatial overlap rather than a categorical one. Values of 0 and 1 correspond to complete exclusion from, and complete embedding within, an annotated domain, respectively, while intermediate values indicate partial overlap. The descriptive labels used in Table 3 (e.g., “Mostly Extra-domain”) summarize that specific finding only and should not be read as general or formally derived classification thresholds.

**Table 3:** MDCS statistics for HIF-1α sequence patterns. The table includes six motif classes associated with experimentally studied regulatory mechanisms. Interpretation labels are descriptive summaries and are not general classification thresholds.

Motifs (ELM class)	Mean MDCS	Interpretation
MOD_SUMO_for_1	0.00	Extra-domain (SLiM-like)
LIG_KEPE_2	0.00	Extra-domain (SLiM-like)
MOD_CDK_SPxK_1	0.00	Extra-domain (SLiM-like)
DEG_ODPH_VHL_1	0.00	Extra-domain (SLiM-like, ODD)
MOD_ProDKin_1	0.21	Mostly Extra-domain
MOD_CK1_1	0.66	Mixed / ambiguous
LIG_LIR_Gen_1	1.00	Domain-embedded (Not SLiM)
DOC_CYCLIN_RevRxL_6	1.00	Domain-embedded (Not SLiM)
LIG_Actin_WH2_2	1.00	Domain-embedded (Not SLiM)

Across the six experimentally supported motif classes, four exhibit mean MDCS values of 0.00, indicating complete exclusion from annotated domains across all observed instances. A fifth motif class shows a low mean MDCS (0.21), while only one motif class displays a higher mean MDCS (0.66). The remaining three motifs show mean MDCS = 1.00, that interprets these motifs are domain-associated rather than canonical SLiMs. These results are summarised in Table 3.

The predominance of low MDCS values among experimentally supported motif classes is consistent with the established localisation of SLiMs in intrinsically disordered regions. For example, phosphorylation sites identified in HIF-1α [27,30,32], SUMOylation sites [28], and interaction motifs [29] are all reported within flexible regions rather than structured domains. Similarly, the VHL-recognition degron characterised structurally by [31] resides within the ODD, which is intrinsically disordered in isolation and only adopts structure upon binding.

The observation that four motif classes show complete exclusion from Pfam domains, and a fifth shows only minimal overlap, therefore aligns with known biological principles governing SLiM localisation. In contrast, the single motif class with elevated MDCS (MOD_CK1_1) is likely influenced by motif degeneracy and Pfam boundary assignment effects. In IDR-rich proteins such as HIF-1α, Pfam annotations may extend into flexible regions, and degenerate motif patterns can generate matches that overlap these annotations.

The intermediate cases (0.21 and 0.66) illustrate the practical value of reporting MDCS as a continuous score. they allow clear-cut instances of exclusion or embedding to be distinguished from ambiguous ones that warrant closer inspection, for example due to motif degeneracy or uncertainty in domain boundary assignment.

Overall, these results demonstrate that low MDCS values are consistent with SLiM-like behaviour in HIF-1α and may serve as a useful descriptive indicator when interpreted alongside biological context.

### Performance evaluation

The performance of ProteoMapper was benchmarked using a large dataset of ADF/cofilin protein sequences and a set of representative motif patterns, including cell cycle docking motifs, MAPK-related motifs, localization signals, and actin-binding signatures. All benchmarking was performed on a single machine equipped with an AMD Ryzen 5 5500U processor (6 cores/12 threads) and 8 GB of 3200 MHz RAM, running Ubuntu 22.04 LTS. The performance table demonstrates efficient scaling with dataset size and number of processes (Table 4).

**Table 4:** Performance scaling of ProteoMapper. Runtime and speedup are shown by sequence count and process number.

Sequences	Processes	Total (s)	Speedup
50	1	12.29	–
100	1	23.51	–
200	1	50.42	–
400	1	98.65	–
800	1	211.82	1.00
800	2	128.02	1.65
800	3	105.58	2.01
800	4	95.23	2.22
800	5	87.79	2.41
800	6	83.90	2.53

To quantify parallel efficiency, we define speedup as:


$$\begin{array}{c} \text{Speedup}=\frac{T_{\text{baseline}}}{T_{n}}, \end{array}$$

where *T*_baseline_ represents the runtime with one process and $T_{n}$ represents the runtime with *n* processes.

Execution time increased approximately linearly with the number of input sequences under single-process conditions, indicating stable scaling behavior for larger datasets. Runtime increased from 12.29 s for 50 sequences to 211.82 s for 800 sequences. Parallel execution reduced runtime for the fixed dataset of 800 sequences. The observed speedup reached 2.53× at 6 processes. However, the gain was sub-linear, with diminishing returns as the number of processes increased, consistent with overhead from task distribution and I/O operations rather than from a hardware ceiling.

Overall, the results suggest that ProteoMapper benefits from parallelization, while maintaining predictable scaling with increasing dataset size. As benchmarks were run on a single 6-core/12-thread consumer laptop, absolute runtimes are expected to improve on higher-core-count or server-class hardware, though the qualitative scaling trends should hold. Input and output datasets of this benchmarking test are available at https://github.com/sifullah0/ProteoMapper/tree/main/Performance%20Benchmarking.

## Discussion

ProteoMapper is designed as a practical framework for alignment-aware visualization and integration of motif and domain annotations. It simplifies analysis that would require multiple separate workflows and manual integration of results. The case studies presented here demonstrate the expected outcome of domain detection, the utility of integrated motif-domain visualization, and the application of positional conservation scoring across protein families. While these analyses reveal quantitative patterns in motif placement and domain context, they are intended to support hypothesis generation rather than provide definitive biological conclusions.

Motifs in this work include both domain-associated sequence signatures and short linear motifs (SLiMs), which differ fundamentally in their structural and functional context. Domain-associated signatures such as PROSITE patterns are diagnostic sequence motifs that define family membership within a folded domain architecture. Their presence within a predicted domain boundary is therefore expected and does not imply an independent regulatory function. In contrast, SLiMs are typically located in intrinsically disordered or flexible regions and mediate transient protein–protein interactions or serve as sites of post-translational modification [4,33]. The biological interpretation of motif–domain overlap therefore requires caution. An observed overlap between a motif pattern and a predicted domain may reflect either a true domain signature or a coincidental match arising from the limitations of pattern-based detection. ProteoMapper does not attempt to resolve this ambiguity automatically but instead provides quantitative descriptors–namely the Motif–Domain Coverage Score (MDCS), positional dispersion (σ), and conservation-threshold classification–to support user interpretation.

A central methodological contribution of this work is the introduction of quantitative metrics that formalize motif–domain relationships in an alignment-aware manner. Positional conservation scoring identifies motif occurrences fixed at identical alignment coordinates across a defined proportion of sequences, providing an explicit measure of evolutionary constraint on motif placement. Unlike frequency-based motif counts, this approach captures positional specificity, which is critical for distinguishing functionally constrained motifs from tolerated sequence patterns. The Motif–Domain Coverage Score (MDCS) provides a descriptive measure of spatial overlap rather than a direct indicator of functional importance. It enables systematic categorization of motif occurrences as domain-embedded, partially overlapping, or extra-domain, supporting comparative analysis across aligned sequences. However, MDCS values should be interpreted in conjunction with biological knowledge and not as standalone evidence of functional relevance. This remains a necessary rather than sufficient condition for SLiM identity, which ultimately requires experimental functional characterisation and demonstration of evolutionary conservation of both the pattern and its disordered sequence context. For example, in the ERD6-like protein analysis, both PS00216 and PS00217 were fully embedded within domains (MDCS = 1.0) but only PS00217 exhibited strict positional conservation. This difference indicates divergence in positional constraints acting on the two signatures across the EDL subfamily, consistent with lineage-specific sequence variation [20], although the functional implications require experimental validation.

ProteoMapper complements, rather than replaces, established tools such as InterProScan, MEME Suite, and SMART, which provide more advanced motif discovery or domain annotation capabilities [21,34,35]. InterProScan remains the most comprehensive platform for large-scale domain annotation through integration of multiple signature databases, but requires command-line expertise, substantial computational resources, and produces complex output formats that are not optimized for interactive interpretation [34]. HMMER is the standard for profile-HMM–based domain detection, yet its text-based outputs require additional downstream processing to support integrative analyses [36]. The primary contribution of ProteoMapper lies in integrating motif detection, domain annotation, and alignment-level visualization into a single, user-friendly workflow with basic quantification of motif-domain overlap and primary categorization of their structural context, particularly for users without programming expertise. A structured comparison of ProteoMapper with established motif and domain analysis tools is provided in S1 Table.

Motif-focused tools address different analytical needs. ScanProsite provides convenient web-based detection of curated PROSITE signatures but does not support domain annotation or large-scale batch analysis [12]. EMBOSS Fuzzpro enables command-line pattern matching but lacks alignment-level visualization and domain-context interpretation [37]. Probabilistic motif detection frameworks such as Wregex offer improved sensitivity for weak or degenerate motifs through weighted regular expressions and position-specific scoring matrices [38,39]. However, these approaches require curated training data and generate continuous scores that are less intuitive to interpret across aligned protein families, and they do not integrate domain annotation or structured visualization. Alignment-based motif detection tools such as MAFin provide conservation-aware motif identification and support multiprocessing at the command line [40], but focus primarily on nucleic acid motifs and do not provide an integrated graphical workflow for protein domain and motif analysis. Other systems emphasize downstream interpretation. CoSMoS ranks motif occurrences by evolutionary conservation but relies on precomputed alignments, lacks domain annotation, and offers limited organism coverage [41]. Resources such as the ELM database, provide motif annotations for individual sequences [9–11], while ProteoMapper enables batch analysis of motif occurrences across multiple aligned homologous proteins simultaneously. This allows consistent positional comparison of motif patterns, which is not directly supported by single-sequence annotation tools. Within this landscape, ProteoMapper provides a practical, MSA-centric interface for integrating motif detection and domain annotation with basic quantification of their spatial relationships, without requiring additional programming expertise.

Sequence-based motif and domain identification tools also remain in active and widespread use despite advances in deep learning-based structure prediction [42]. Structure-prediction methods provide powerful three-dimensional context, but they do not remove the need for rapid sequence-level screening, protein-family comparison, or annotation of conserved sequence features. This distinction is particularly relevant for short linear motifs, such as those examined in the HIF-1α case study, because many SLiMs occur in intrinsically disordered or flexible regions and are not always straightforwardly interpreted from a single predicted folded structure. ProteoMapper should therefore be viewed as operating alongside structural workflows, addressing a complementary rather than substitutable analytical need, rather than as a competing approach.

Several limitations of the current framework should be acknowledged. Motif detection is based on regular expressions, which do not capture probabilistic sequence variation and may produce false positives compared to methods based on position-specific scoring matrices or machine learning approaches. Domain boundaries are derived from sequence-based HMM predictions using HMMER, which may not correspond precisely to structural domain boundaries as defined by structure-based approaches such as AlphaFold-derived domain decomposition approaches [13,14]. The MDCS metric may be influenced by false positives in both motif detection and domain boundary prediction, potentially leading to overestimation of motif–domain embedding. Degenerate short patterns may match by chance within domain regions, and HMMER domain boundary coordinates represent sequence-level HMM alignment endpoints rather than precise structural boundaries, both of which may affect the accuracy of MDCS classification. The current implementation does not incorporate intrinsic disorder prediction, which is important for interpreting SLiMs and distinguishing functional regulatory motifs from structural sequence patterns [43]. These limitations indicate that ProteoMapper is best suited for exploratory and hypothesis-generating analyses, and its results should be interpreted alongside complementary computational or experimental evidence.

Future development will focus on integrating probabilistic motif scoring approaches, incorporating disorder prediction to better interpret SLiMs, and improving compatibility with structural annotation pipelines. Although ProteoMapper produces Excel workbooks to support interactive visualization, FASTA input is fully supported for standard sequence-analysis workflows. Excel input and output are retained primarily to make alignment inspection and annotated results accessible to users who prefer graphical and spreadsheet-based workflows. Future versions may include additional plain-text or Markdown summary outputs to improve compatibility with lightweight, command-line-associated analysis pipelines. These extensions aim to broaden analytical scope without compromising accessibility or alignment-aware visualization.

In summary, ProteoMapper does not replace specialized tools for motif discovery or domain annotation. Its primary contribution lies in the alignment-aware integration of motif detection, domain annotation, and positional conservation within a unified platform, enabling users to examine positional relationships between sequence features in a clear biological context. ProteoMapper provides a computational filter in a multi-step process of SLiM candidate prioritisation.

## Conclusion

ProteoMapper provides a user-friendly framework for integrating motif detection, domain annotation, and alignment-based conservation analysis within a unified desktop environment. The tool accepts both Excel and FASTA input formats and produces structured spreadsheet outputs with alignment-level visualization, supporting exploratory analysis for users without programming expertise. Validation across protein families demonstrated consistency with published domain annotations. In PLATZ transcription factors and actin-depolymerizing factors, domain detection showed high overlap with established annotations (mean intersection-over-union of 0.94 for BrPLATZ and 100% detection of ADF-H domains). In the ERD6-like transporter analysis, both PROSITE signatures PS00216 and PS00217 showed full domain embedding (MDCS = 1.0), yet only PS00217 exhibited strict positional conservation across sequences. This pattern may warrant further investigation through structural or experimental approaches, though such interpretations require validation beyond computational analysis alone. Similarly, the HIF-1α case study demonstrated that MDCS can distinguish known regulatory motifs localized to disordered regions (mean MDCS = 0.00) from domain-associated patterns, consistent with established principles of short linear motif localization. ProteoMapper complements specialized resources such as HMMER, ScanProsite, and the MEME Suite [12,35,36] rather than replacing them. The primary strength lies in reducing technical barriers to exploratory analysis, particularly for experimental researchers examining conserved motifs and domain architectures in protein families. By enabling direct comparison of motif occurrences, positional conservation, and domain context within a single output file, the framework supports exploratory analysis and the formulation of hypotheses related to regulatory mechanisms, evolutionary constraints, and functional organization. Several limitations should also be noted. Motif detection relies on regular expressions, which may produce false positives compared to probabilistic approaches. Domain boundaries are derived from sequence-based HMM predictions and may not correspond to structural domain definitions. MDCS values do not distinguish functional relevance from coincidental overlap and should be interpreted alongside biological knowledge. The current implementation does not incorporate intrinsic disorder prediction, which is important for contextualizing short linear motifs. Future development will focus on integrating probabilistic motif scoring while preserving interpretability, incorporating disorder prediction to better distinguish functional SLiMs, and improving compatibility with structural annotation pipelines. In summary, ProteoMapper offers a practical solution for combining motif detection, domain annotation, and conservation analysis within an alignment-centric workflow. It supports reproducible exploratory analysis of protein architecture and evolutionary constraint, particularly for users seeking to examine spatial relationships between sequence features without extensive bioinformatics infrastructure. It provides an accessible environment for integrating their outputs into interpretable, visually structured formats suitable for comparative protein studies.

## Supporting information

S1 Table Comparative feature analysis of ProteoMapper and established motif/domain analysis tools. This table compares ProteoMapper with InterProScan, ScanProsite, Wregex, MAFin, MEME Suite, and GOmotif across key software features.
(DOCX)
