# Supplementary material for: ProteoMapper: Alignment-aware identification and quantitative analysis of contextual motif–domain patterns in protein families
Source: PLoS One. 2026 Aug 10;21(8):e0348861. doi: 10.1371/journal.pone.0348861 (PMC13456289; doi:10.1371/journal.pone.0348861)
Supplement: Supplementary file 1 [file pone.0348861.s001.docx]

# Comparative Feature Analysis of Motif and Domain Analysis Tools

| Feature | ProteoMapper | InterProScan | ScanProsite | Wregex | MAFin | MEME Suite | GOmotif |
| --- | --- | --- | --- | --- | --- | --- | --- |
| GUI (Desktop / No-Code) | Yes (Excel I/O) | No (CLI / Web) | Yes (Web) | No (CLI / Web) | No (CLI) | Yes (Web) | Yes (Web) |
| Custom Motifs | Yes (Regex) | No | Yes (PROSITE) | Yes (Weighted Regex) | Yes (Regex / Alignment-based) | No (Discovery) | No |
| Domain Scanning | Yes (HMMER / Pfam) | Yes (Multi-DB) | No | No | No | No | No |
| Multi-processing | Yes (User-set cores) | Yes (CLI -cpu) | No | No | Yes (CLI ) | Limited | No |
| Conservation Insights | Yes (Alignment-level marking) | No | No | No | Yes (Alignment-based frequency) | Limited (Enrichment) | No |
| Motif–Domain Insights | Yes (MDCS) | Limited (Positional overlap) | No | No | No | No | GO-linked motifs |
| Output | Excel (Colors, Borders, MDCS) | XML / TSV / HTML | HTML / Text | Text | Text / CSV | HTML (Motifs) | HTML (GO associations) |
